# The difference in hemodynamic responses between dominant and non-dominant hands during muscle contraction and relaxation: An fNIRS study

**DOI:** 10.1371/journal.pone.0220100

**Published:** 2019-07-19

**Authors:** Naoko Yokoyama, Chiaki Ohtaka, Kouki Kato, Hiroko Kubo, Hiroki Nakata

**Affiliations:** 1 Faculty of Human Life and Environment, Nara Women’s University, Nara City, Japan; 2 Faculty of Sport Sciences, Waseda University, Tokorozawa, Japan; LUNEX International University of Health, Exercise and Sports, LUXEMBOURG

## Abstract

The present study used functional near-infrared spectroscopy (fNIRS), and investigated the differences in neural activation of ipsi- or contralateral hemispheres between right dominant and left non-dominant hands among right-handed subjects using consecutive motor tasks with muscle contraction and relaxation. The subjects performed tasks under four conditions: (1) right hand up (R-Up), (2) left hand up (L-Up), (3) right hand down (R-Down), and (4) left hand down (L-Down). The peak amplitude of oxy-Hb was significantly larger at the contralateral than ipsilateral hemisphere in the premotor area (PM) under the R-Up condition, and no significant differences were observed between contra- and ipsilateral hemispheres under the L-Up condition. In addition, the peak amplitude was more negative at the contra- than ipsilateral hemisphere in the PM under the R-Down condition, while the peak amplitude was significantly more negative at the ipsi- than contralateral hemisphere in the PM under the L-Down condition. These results suggest that the PM of the left hemisphere among right-handed subjects plays an important role in muscle contraction and relaxation with force control.

## Introduction

Adequate motor control is essential for our daily life and sports activities. Unilateral movement is basically controlled by the primary motor cortex (M1) in the hemisphere contralateral to the moving hand or foot. However, recent neuroimaging and neurophysiological studies using functional magnetic resonance imaging (fMRI) and transcranial magnetic stimulation (TMS) noted that neural activation in the ipsilateral M1 during unilateral hand movement is often greater in the left hemisphere (i.e., the left M1) than right hemisphere (i.e., the right M1) among right-handed subjects [1–4]. This indicates that asymmetric neural activity in the M1 is involved in motor execution, and the left hemisphere is specialized for movement control of either hand in right-handed subjects [5]. In addition, Verstynen and colleagues [6] showed that neural activation in the ipsilateral M1 was higher during complex movement tasks than during a simple movement task. These studies suggest that recruitment of neural activation in the ipsilateral M1, especially in the left M1 for right-handed subjects, is related to the difficulty of required motor tasks. This asymmetric activation is not only observed in the M1 but also in other areas of the left hemisphere, particularly in the premotor (PM) and posterior parietal cortices (PPC) [7, 8].

The voluntary movements involve some aspects including motor preparation, response selection, execution, and inhibition [9]. Muscle relaxation is also one of the important factors for fine motor control. Our previous studies using behavioral data showed that force control is more difficult in muscle relaxation than in muscle generation [10]. Some previous studies using fMRI, TMS, and electroencephalography (EEG) reported that neural activation in the M1, supplementary motor area (SMA), PM, dorsolateral prefrontal cortex (DLPFC), and anterior cingulate cortex (ACC) was associated with muscle relaxation [11–14]. However, to the best of our knowledge, no previous studies focused on the differences in neural activation of ipsi- or contralateral hemispheres between dominant and non-dominant hands during muscle relaxation. Therefore, it remains unclear whether the neural substrate for muscle relaxation differs between dominant and non-dominant hands, even though neuroimaging studies reported the brain regions associated with muscle relaxation.

In the present study, we aimed to clarify this using functional near-infrared spectroscopy (fNIRS). fNIRS is a tool for noninvasively monitoring the tissue oxygenation and hemodynamics of the human brain. The characteristics of hemodynamic responses during motor execution and imagery have been reported by utilizing fNIRS [15–17]. However, the characteristics of hemodynamic responses during motor relaxation using fNIRS are poorly understood. In order to investigate the hemodynamic responses for both muscle contraction and relaxation, we employed consecutive motor tasks including muscle contraction and relaxation with force control. Daily life movement performances (e.g., gripping objects) as well as sports-specific movement techniques require combined and more complex voluntary movements rather than isolated muscle contractions or relaxations or complete rest. Recently, Vogt and colleagues [18] used consecutive motor tasks with muscle contractions (40% MVC) and relaxations (20% MVC), and showed that the amplitudes of motor-related cortical potentials obtained by time-locked averaging EEG were larger on muscle contraction than muscle relaxation at frontal electrode sites. The present study followed the protocol of Vogt’s study.

As a result, asymmetric neural activation between right-dominant and left non-dominant hands among right-handed subjects was observed during muscle contraction and relaxation. The present study provides evidence for the underlying neural mechanisms of muscle contraction and relaxation in the study field of motor control.

## Materials and methods

### Subjects

Twenty normal right-handed females (mean age: 21.6 years, range: 20–23 years) participated in this study. All subjects were undergraduate or graduate students. They were all right handed according to the criteria of the Edinburgh Inventory [19], and the mean score was 90±9. The subjects had no record of neurological or psychiatric disorder. The subjects were informed in detail about the experiments prior to participation, and gave their informed consent for involvement in this study. The study was approved by the Ethics Committee of Nara Women’s University, Nara, Japan. Experiments were conducted in accordance with the Declaration of Helsinki.

### Procedure

The experiment was performed in a quiet room maintained at 24°C. Recordings were conducted under four conditions: (1) right hand up (R-Up), (2) left hand up (L-Up), (3) right hand down (R-Down), and (4) left hand down (L-Down). At the beginning of the experiment, the grip strength of the each hand was measured using an electronic hand dynamometer (TKK5710b, Takei Scientific Instruments Co., Ltd. Japan). The dynamometer was adjusted to best fit the grip of the subject’s hand. Then, the subject sat comfortably on a chair, and the dynamometer was placed on a table in front of them. The subject was asked to squeeze the grip as hard as possible for 2 sec without moving their arm, and was verbally encouraged to maximize the force. They performed this action three times, and the maximal value among the three trials was adopted as the subject’s maximum voluntary contraction (MVC). After this determination, 10, 20, and 30% MVC of the grip strength were calculated.

**Fig 1:**
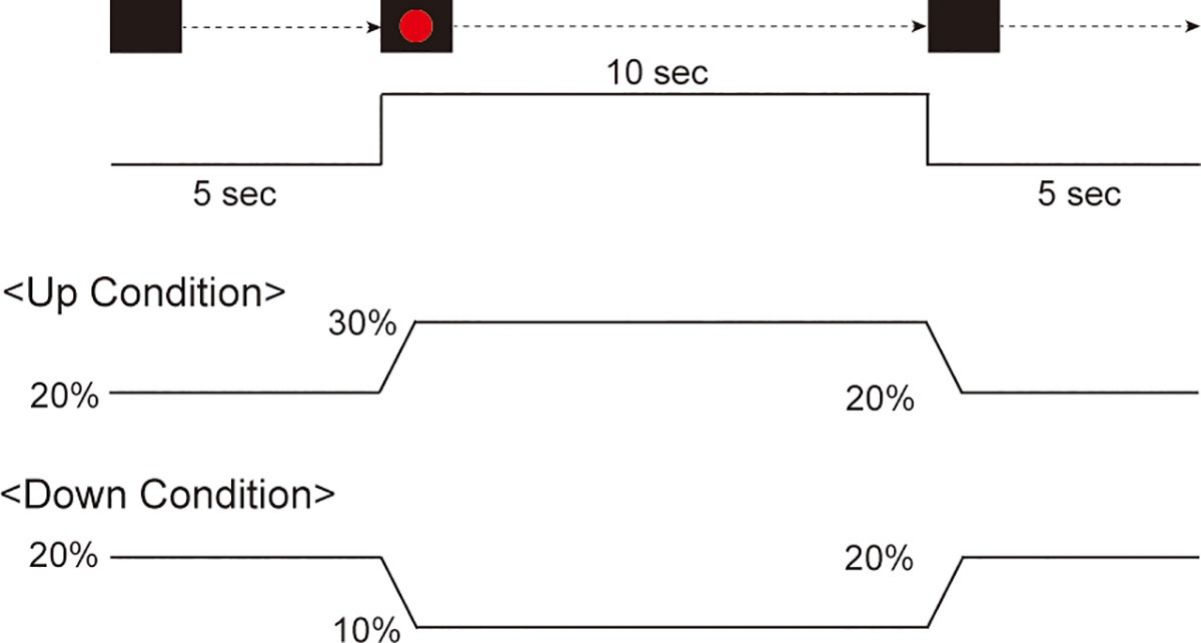
Simplified scheme of experimental design. The subject was asked to match the target line with 20% MVC and the force trajectory line for 5 sec with no signal. (Up condition) When a red signal was presented for 10 sec, the subject was instructed to match the target line with 30% MVC and the force trajectory line for 10 sec. (Down condition) During the same period, the subject was instructed to match the target line with 10% MVC and the force trajectory line for 10 sec. After presenting a red signal, the subject was asked to match the target line with 20% MVC and the force trajectory line for 5 sec under both conditions.

In the R-Up and L-Up conditions, at the beginning of each trial, the subject was asked to adjust the force level so as to match the target line representing 20% of their MVC and the force trajectory line as accurately as possible for 5 sec. The force target and trajectory lines were displayed on a monitor in front of the subject at a distance of approximately 1 m. Then, a red signal was presented for 10 sec, and the subject was instructed to match the target line with 30% MVC and the force trajectory line as accurately and quickly as possible for 10 sec. When eliminating a red signal, the subject was asked to match the target line with 20% MVC and the force trajectory line for 5 sec (Fig 1). They were asked to repeatedly perform this for ten trials. In the R-Down and L-Down conditions, at the beginning of each trial, the subject was instructed to match the target line with 20% MVC and the force trajectory line as accurately as possible for 5 sec. Then, a red signal was presented for 10 sec, and they were instructed to match the target line with 10% MVC and the force trajectory line as accurately and quickly as possible for 10 sec. They were asked to repeatedly perform this for ten trials. A practice session of several trials for each movement was performed before the recording to enable subjects to become familiar with the experimental situation. A 5-min break was set to avoid the effects of fatigue after each condition. The order of the four conditions was randomized for each subject and counterbalanced across all subjects.

**Fig 2:**
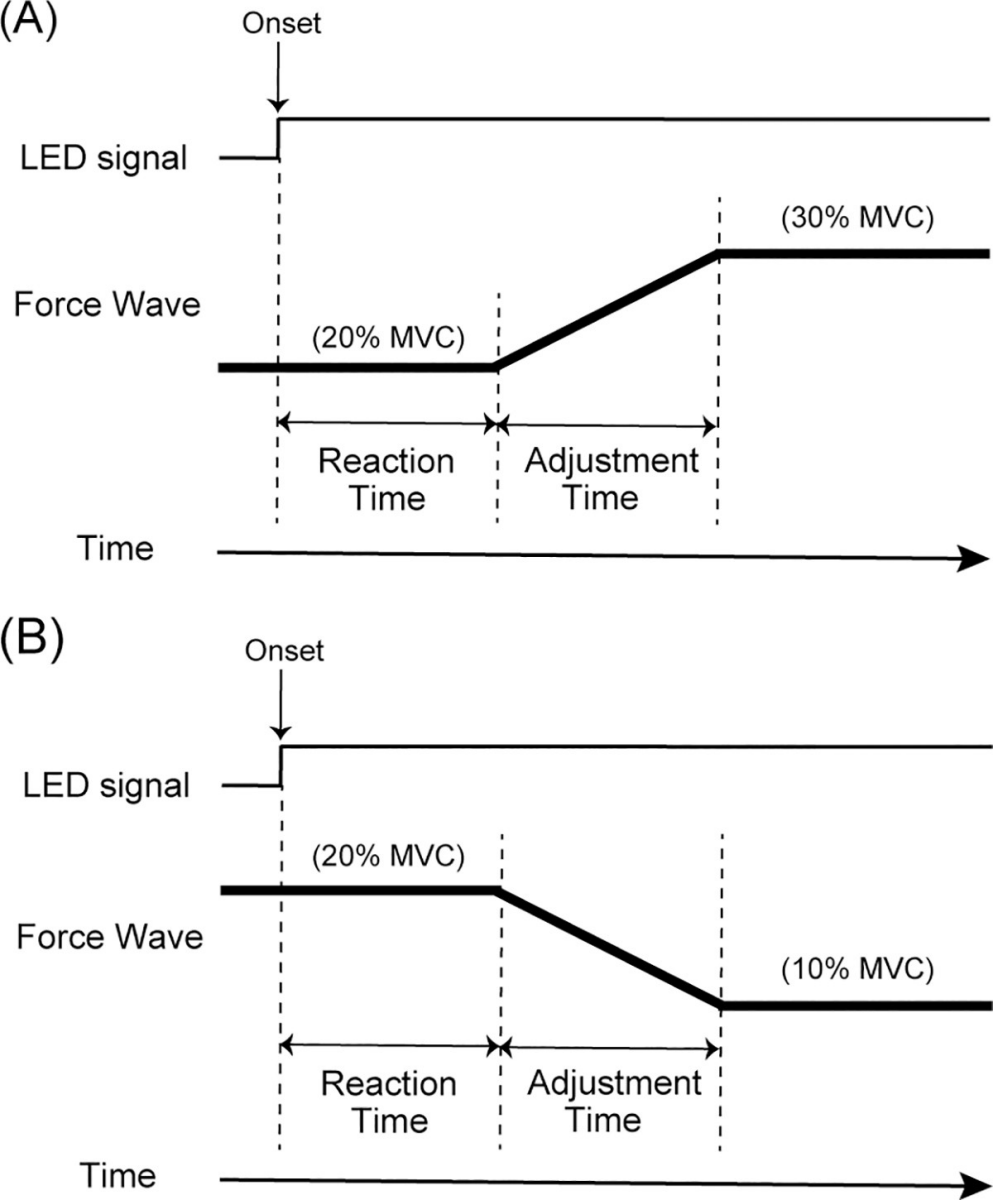
Definition and measurement of force under (A) Up and (B) Down conditions.

The produced force was recorded. All of the recordings were digitized at 1,000 Hz using a Biopac MP150 data acquisition system (Biopac Systems, USA). The force data were passed through a digital filter with a 100-Hz low-pass cutoff. Fig 2 shows the definition and measurement of force, following previous studies [10, 20]. The average of the rate of force development (N/s) of the 10-ms period after the LED signal was calculated. The baseline was the force average of the 300-ms period before the LED signal. Subsequently, the force onset was defined as the first time point of the first period during which the average of the rate of force development exceeded 50% more than the Up condition and was less than -50% more than the Down condition. The reaction time was defined as the time between the LED signal and force onset. The reaction time is an important measure for understanding sensorimotor performance in humans [21], defined as the time from the stimulus onset to response. The adjustment time was defined as the time between the force onset and the maximum force under the Up condition (Fig 2A) or minimum force under the Down condition (Fig 2B). The adjustment time is an index of the accuracy and speed to achieve the target force level [10, 20]. The timing of the maximum and minimum forces was determined between the force onset and maintained force period.

### NIRS measurement

**Fig 3:**
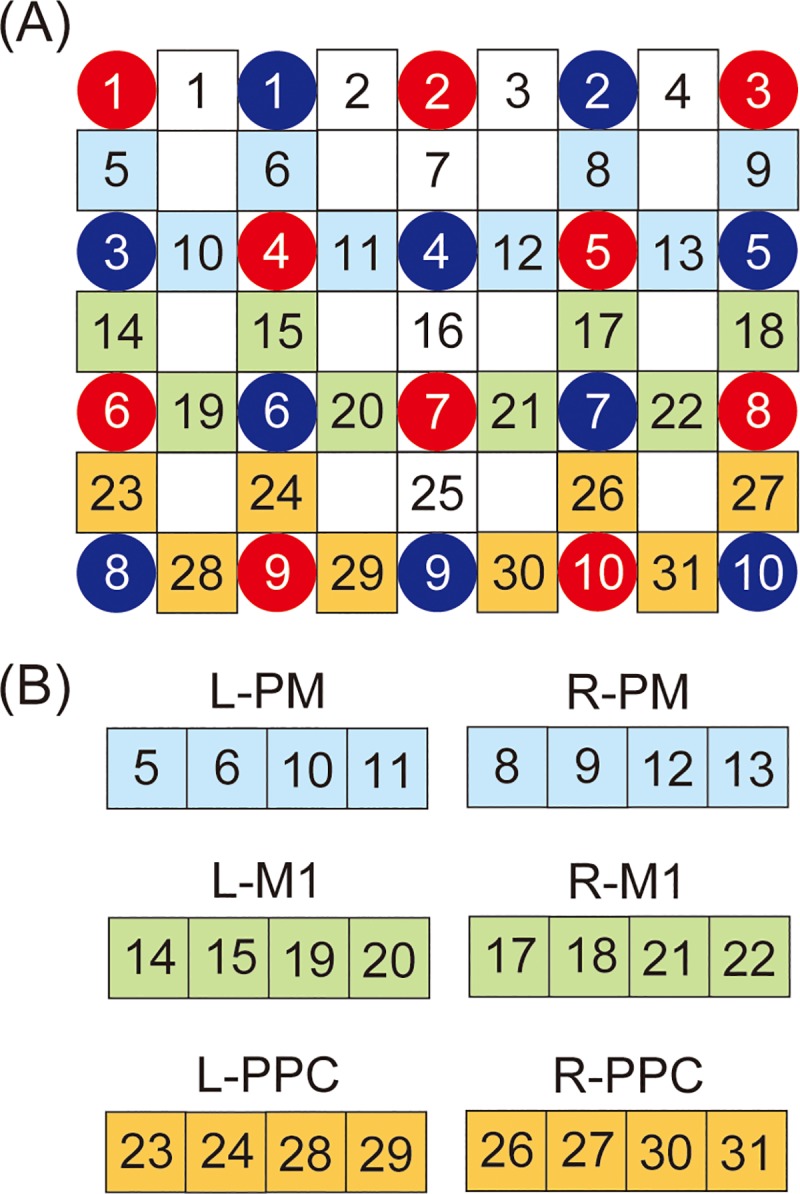
(A) Channel configuration of the optical probes. Ten red and blue circles indicate the positions of NIRS sensors and detectors, respectively, in 31 optical channels (5 x 4 optical probe set). (B) The defined regions of interest (ROIs). L-PM = left premotor cortex; R-PM = right premotor cortex; L-M1 = left primary motor cortex; R-M1 = right primary motor cortex; L-PPC = left posterior parietal cortex; R-PPC = right posterior parietal cortex.

The hemoglobin concentrations were measured at sampling of 160 ms using the NIRS system (FOIRE-3000, Shimadzu Corporation, Kyoto, Japan). The absorption was measured at three wavelengths of near infrared light: 780, 805, and 830 nm, and the separation distance between the source and detector was 3.0 cm. The optical probes consisted of 10 sources and 10 detectors, resulting in a total of 31 channels (5 x 4 optical probe set) (Fig 3A). The NIRS probes were attached according to the international 10–20 electrode system employed in EEG, and the Cz position was used as a marker for ensuring replicable placement of the optodes [15]. In the present study, the Cz was placed at channel 7 of the source. This probe position was followed by previous studies investigating motor-related areas [15, 17, 22, 23]. The regions of interest (ROIs) were defined as follows: channels 5, 6, 10, and 11 in the left hemisphere and channels 8, 9, 12, and 13 in the right hemisphere corresponding to PM; channels 14, 15, 19, and 20 in the left hemisphere and channels 17, 18, 21, and 22 in the right hemisphere corresponding to M1; channels 23, 24, 28, and 29 in the left hemisphere and channels 26, 27, 30, and 31 in the right hemisphere corresponding to PPC. Channels 1, 2, 3, 4, 7, 16, and 25 were not further analyzed (Fig 3B).

**Fig 4:**
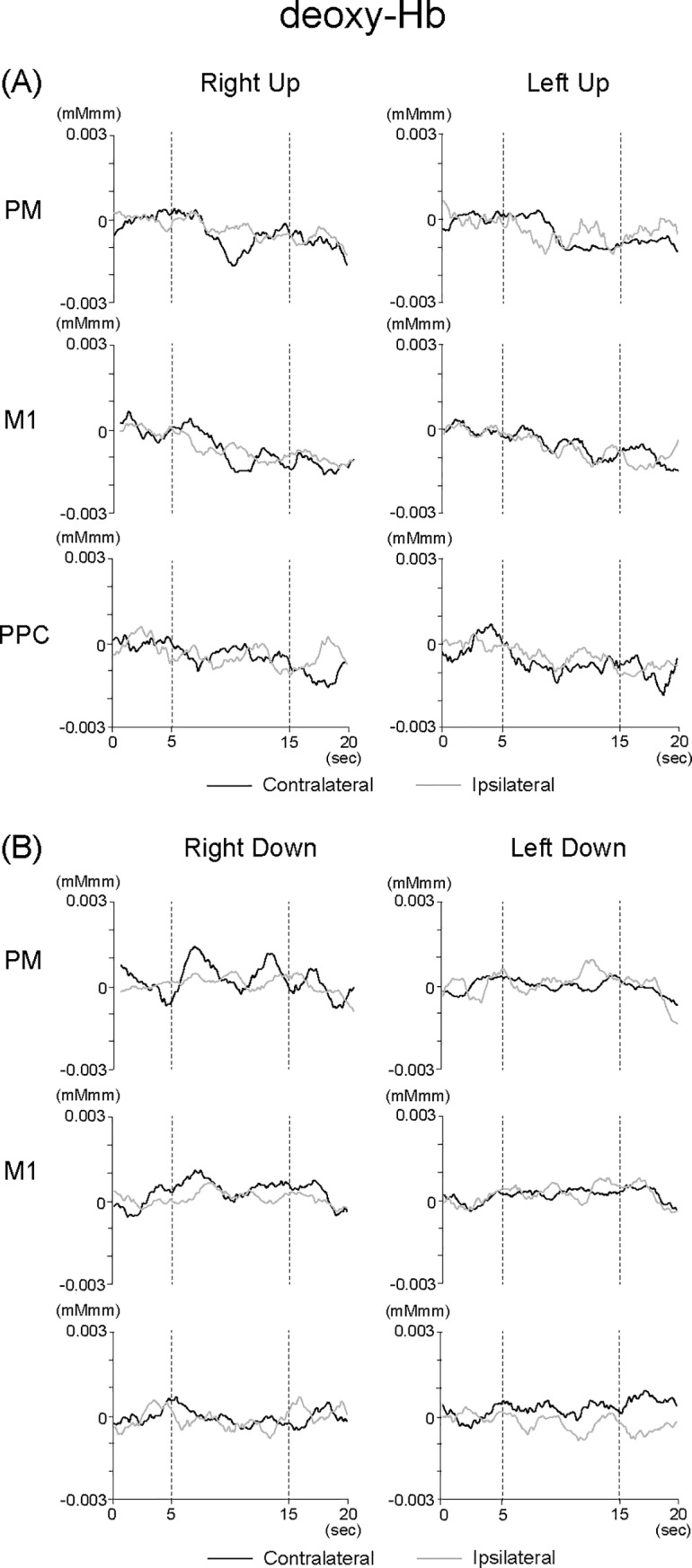
Grand-averaged waveforms of deoxy-Hb across all subjects in contra- and ipsilateral hemispheres in the PM, M1, and PPC under (A) Up and (B) Down conditions.

The recorded changes in light intensity were converted into changes of optical density. Afterward, we used the modified Beer-Lambert law to convert changes in optical density into concentration changes of oxygenated (oxy-Hb) and deoxygenated hemoglobin (deoxy-Hb). Similar to previous studies, no specific value of differential path length factor was used, and thus the scale unit of the measured values was molar concentration multiplied by unknown path length factor (mMmm) [16, 24]. Then, the moving average was calculated, and baseline normalization was conducted. The moving average was used to reduce noise and longitudinal signal drift with a smoothing factor of twenty-five points, based on previous studies [15, 22, 25], which was consistent with a low-pass filter set at 0.11 Hz. The signal changes were referenced to a baseline interval of -5 to 0 sec. We averaged ten trials at each channel, and then calculated the signal changes in each ROI by averaging over four channels. As for oxy-Hb concentration changes in each ROI, the peak amplitude and latency were determined with the baseline-to-peak measurement during R-Up, L-Up, R-Down, and L-Down conditions. The baseline-to-peak measurement was useful to identify the amount and timing of neural activity. As for deoxy-Hb concentration changes in each ROI, clear peaks were not always determined (Fig 4). Therefore, we report the results based on oxy-Hb concentration changes.

### Analyses

Data on four subjects under the Up condition were excluded because fNIRS data included unexplained noise with a signal drift in an inverse direction. Data on four other subjects under the Down condition were also excluded for the same reason. Thus, the data on sixteen subjects were separately analyzed under the Up and Down conditions. As behavioral data, we calculated the mean values and variability (standard deviations) of the reaction time and adjustment time for all participants during all conditions (Fig 2). Then, data were separately submitted to two-way analysis of variance (ANOVA) using Hand (left vs. right) and Condition (Up vs. Down) factors.

Data on the peak amplitude and latency under the Up conditions were separately submitted to two-way ANOVA with repeated measures using Hand and Laterality (contralateral vs. ipsilateral hemisphere) as within-subjects factors. Data on the peak amplitude and latency under the Down conditions were also analyzed with the same repeated measures ANOVA. Significance was set at p < 0.05.

## Results

### Behavioral data

Two-way ANOVA for the reaction time showed a significant main effect of Condition (F (1, 60) = 5.903, p < 0.05, η^2^ = 0.090), indicating that the reaction time was significantly longer under the Down condition than Up condition. The same ANOVA for the adjustment time revealed a strong tendency for the main effect of Hand (F (1, 60) = 3.335, p = 0.073, η^2^ = 0.053), indicating that the reaction time was later under the Left than Right hand.

ANOVA for variability of the reaction time demonstrated no significant main effects or interaction. ANOVA for variability of the adjustment time showed a significant main effect of Hand (F (1, 60) = 6.636, p < 0.05, η^2^ = 0.100), indicating that the variability of the adjustment time was larger under the Left than Right hand. The mean values and variability for each condition are listed in Table 1.

**Table 1:** Behavioral data for each condition.

	Reaction Time		Adjustment Time	
(ms)	Left hand	Right hand	Left hand	Right hand
Mean value				
Up condition	286 (16)	258 (15)	476 (37)	397 (30) #
Down condition	327 (17) *	299 (19) *	474 (38)	422 (38) #
Variability				
Up condition	67 (8)	69 (11)	249 (24)	201 (24) *
Down condition	101 (15)	89 (27)	335 (45)	218 (29) *

Significant differences in mean value of reaction time between Up and Down conditions are shown as *: p < 0.05. Strong tendencies in mean value of adjustment time between Left and Right hands are shown as #: p = 0.073. Significant differences in variability of adjustment time between Left and Right hands are shown as *: p < 0.05. Data are expressed as the mean with SE.

### fNIRS data

**Fig 5:**
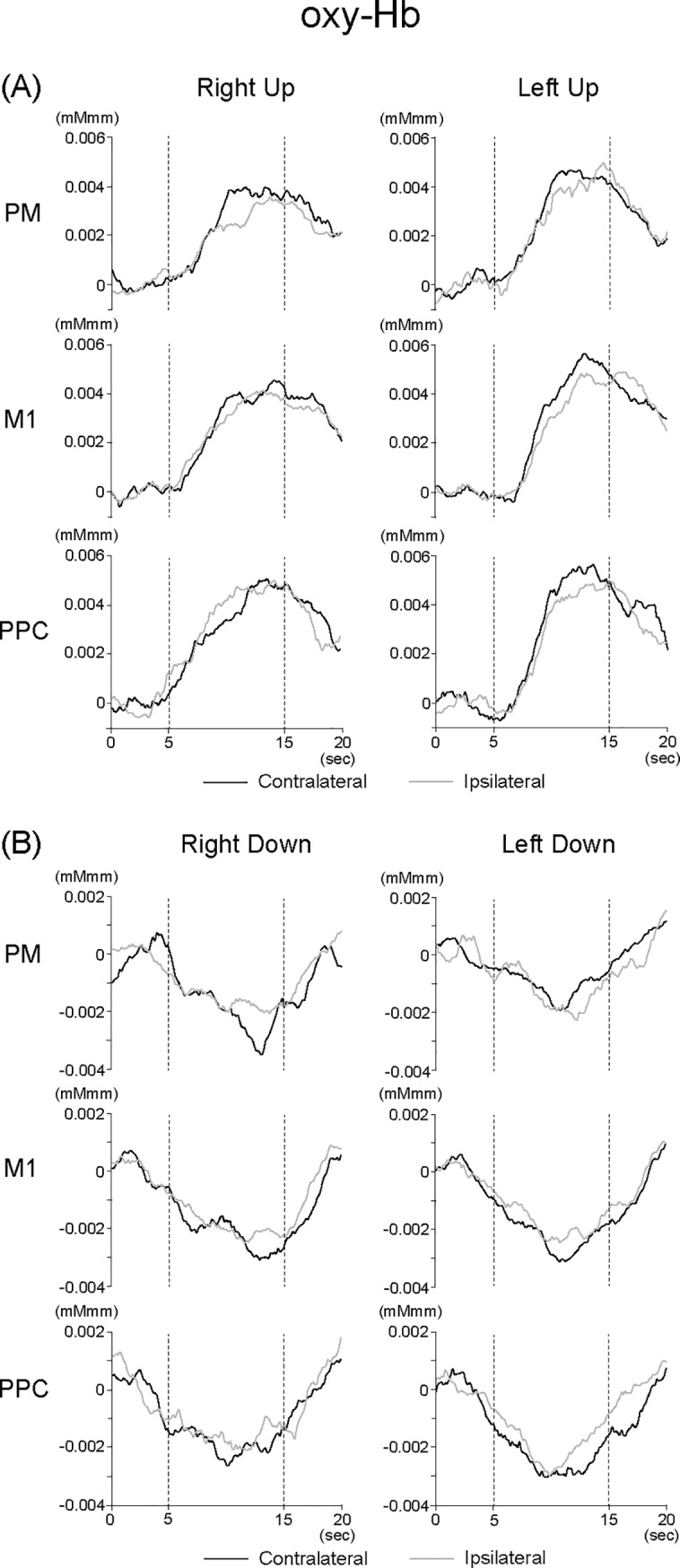
Grand-averaged waveforms of oxy-Hb across all subjects in contra- and ipsilateral hemispheres in the PM, M1, and PPC under (A) Up and (B) Down conditions.

Fig 5 shows grand-averaged waveforms of oxy-Hb across all subjects in contra- and ipsilateral hemispheres in the PM, M1, and PPC.

**Fig 6:**
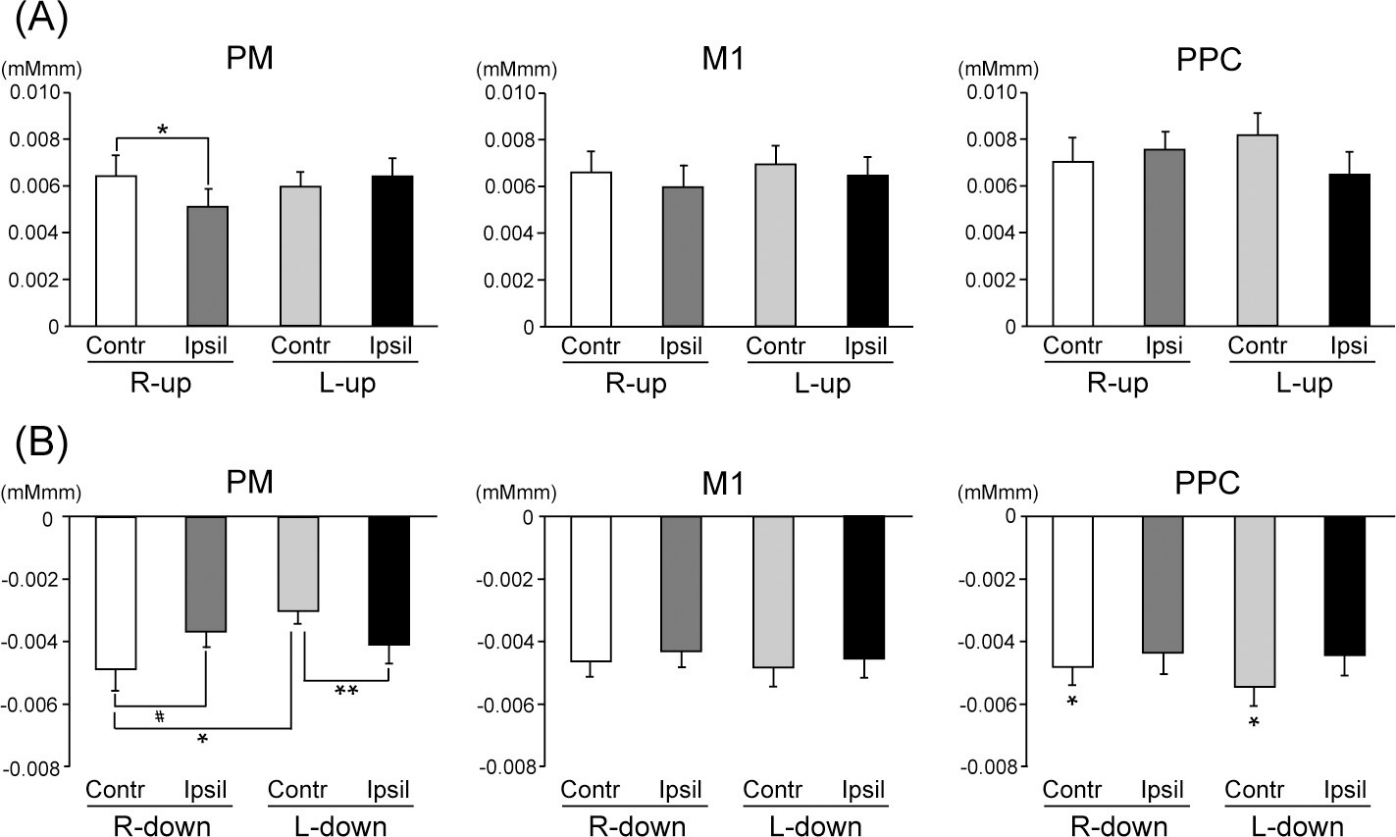
Mean values for peak amplitudes of oxy-Hb in contra- and ipsilateral hemispheres in the PM, M1, and PPC under (A) Up and (B) Down conditions. Significant differences among tasks are shown as * p < 0.05, and ** p < 0.01. Strong tendency toward a difference is shown as # p < 0.07. Data are expressed as the mean with standard error (SE).

ANOVAs for peak amplitudes under the Up condition showed a significant Hand-Laterality interaction in the PM (F (1, 15) = 9.917, p < 0.01, η^2^ = 0.398). Further analyses of Laterality showed that the peak amplitude in the PM was significantly larger in the contra- than ipsilateral hemisphere under the R-Up condition (F (1, 15) = 7.883, p < 0.05, η^2^ = 0.344). No significant main effect or interaction was observed in the M1 and PPC (Fig 6A).

ANOVAs for peak amplitudes under the Down condition showed a significant Hand-Laterality interaction in the PM (F (1, 15) = 9.056, p < 0.01, η^2^ = 0.376). Further analyses of Laterality showed that the peak amplitude in the PM was significantly more negative in the ipsi- than contralateral hemisphere under the L-Down condition (F (1, 15) = 11.305, p < 0.01, η^2^ = 0.430). On the other hand, a stronger tendency was observed whereby the peak amplitude in the PM was more negative in the contra- than ipsilateral hemisphere under the R-Down condition (F (1, 15) = 3.840, p < 0.07, η^2^ = 0.204). Further analyses of Hand showed that the peak amplitude in the PM was significantly more negative under the R-Down than L-Down condition in the contralateral hemisphere (F (1, 15) = 7.513, p < 0.05, η^2^ = 0.334). ANOVAs for peak amplitudes under the Down condition showed a significant main effect of Laterality in the PPC (F (1, 15) = 5.578, p < 0.05, η^2^ = 0.271), indicating that the peak amplitudes were significantly more negative in the contra- than ipsilateral hemisphere. No significant main effect or interaction was observed in the M1 (Fig 6B).

ANOVAs for peak latencies under the Up condition showed a significant Hand-Laterality interaction in the PPC (F (1, 15) = 6.398, p < 0.05, η^2^ = 0.299). Further analyses of Laterality showed that the peak latency in the PPC was significantly shorter in the contra- than ipsilateral hemisphere under the L-Up condition (F (1, 15) = 6.574, p < 0.05, η^2^ = 0.305). No significant main effect or interaction was observed in the PM and M1 (Table 2).

**Table 2:** Peak latency of oxy-Hb for each condition.

	Right Up		Left Up		Right Down		Left Down	
(sec)	Contr	Ipsil	Contr	Ipsil	Contr	Ipsil	Contr	Ipsil
PM	7.74 (0.76)	8.26 (0.68)	7.72 (0.52)	8.03 (0.51)	6.77 (0.43)	7.34 (0.56)*	6.70 (0.59)	7.68 (0.57)*
M1	7.94 (0.67)	7.65 (0.57)	7.80 (0.57)	9.17 (0.60)	6.85 (0.69)	6.85 (0.62)	6.86 (0.62)	7.22 (0.52)
PPC	8.03 (0.68)	7.66 (0.61)	7.45 (0.59)	8.66 (0.56)*	7.16 (0.70)	7.30 (0.63)	6.40 (0.60)	5.83 (0.62)

Significant differences between contra- and ipsilateral hemispheres are shown as *: p < 0.05. Data values are expressed as the mean with SE. Contr = hemisphere contralateral to the moving hand; Ipsil = hemisphere ipsilateral to the moving hand.

ANOVAs for peak latencies under the Down condition showed a significant main effect of Laterality in the PM (F (1, 15) = 6.209, p < 0.05, η^2^ = 0.293), indicating that the peak latency in the PM was significantly shorter in the contra- than ipsilateral hemisphere, irrespective of Hand. No significant main effect or interaction was observed in the M1 and PPC (Table 2).

## Discussion

The present study investigated the differences in neural activation of ipsi- or contralateral hemispheres between right dominant and left non-dominant hands among right-handed subjects using consecutive motor tasks with muscle contraction and relaxation. As behavioral data, we noted differences in the mean values for the reaction time between the Up and Down conditions, and in the variability for the adjustment time between right dominant and left non-dominant hands. The characteristics of the peak amplitude of oxy-Hb in the PM were also clearly different between right dominant and left non-dominant hands under the Up and Down conditions.

We previously examined motor control strategies for accurate force generation and relaxation using graded tasks during isometric force control with the right arm [10]. We found that the errors for 20 and 40% magnitudes of maximum voluntary force were higher for force relaxation than for force generation. The present study, using consecutive motor tasks, also showed a longer reaction time under the Down than Up condition (Table 1). Thus, taking the previous and present findings into consideration, motor control strategies might differ between muscle contraction and relaxation. Furthermore, we found that the variability in the adjustment time was larger under the Left than Right hand (Table 1), indicating that motor control with the left non-dominant hand was more difficult than that with the right dominant hand among right-handed subjects. This characteristic is consistent with many previous studies investigating the characteristics of dominant and non-dominant hands (see a review, [26]).

Some previous studies utilizing fMRI found that neural activation in the M1, SMA, and DLPFC, and neural deactivation in the ACC were associated with muscle relaxation [12, 13]. For example, Toma and colleagues [12] reported that the increase of the blood oxygen level-dependent (BOLD) signal using fMRI was observed after muscle relaxation in the contralateral M1 and bilateral SMA. Spraker and colleagues [13] also found that right DLPFC had exhibited greater activity with a relaxing force compared with a generating force. However, in the present study, oxy-Hb was decreased after muscle relaxation in the bilateral PM, M1, and PPC (Figs 5 and 6). We considered two possible hypotheses for this discrepancy. The first hypothesis involves differences in the required tasks between previous studies and our study. Previous fMRI studies used completely muscle relaxation tasks (e.g., from 15%MVC to 0%), whereas the present study applied muscle relaxation with force control (i.e., from 20 to 10% MVC). The second hypothesis involves a difference in methodology. fMRI studies generally evaluate BOLD signals that reflect a change in the ratio of oxy-Hb to deoxy-Hb, while fNIRS quantifies the changes in both oxy-Hb and deoxy-Hb. Therefore, the modulation as a hemodynamic response might not always be consistent.

The main goal of the present study was to clarify the differences in neural activation of ipsi- or contralateral hemispheres between right dominant and left non-dominant hands among right-handed subjects. Our results showed that the peak amplitude was significantly larger at the contra- than ipsilateral hemisphere in the PM under the R-Up condition, and no significant differences were observed between contra- and ipsilateral hemispheres under the L-Up condition (Fig 6A). This finding is consistent with previous studies using fMRI [1–4], indicating asymmetric activation in the motor-related cortical areas among right-handed subjects. In addition to this, our data showed that the peak amplitude was more negative at the contra- than ipsilateral hemisphere in the PM under the R-Down condition, while the peak amplitude was significantly more negative at the ipsi- than contralateral hemisphere in the PM under the L-Down condition (Fig 6B). Based on these findings, we considered that the PM of the left hemisphere among right-handed subjects played an important role in muscle relaxation as well as contraction with force control. In other words, combined and complex voluntary movements in daily life and sports-specific movements might be related to neural activity in the PM.

We need to consider why the PM was sensitive to muscle relaxation with force control rather than the M1 and PPC. Haaland and colleagues [7] reported that greater left hemisphere activation was observed during complex finger tasks in the PM, PPC, thalamus, and cerebellum, but not in the M1 among right-handed subjects. Moreover, brain-damaged patients showed greater ipsilateral motor impairment after left compared with right hemisphere damage [27], and demonstrated sequencing deficits characterized by impaired planning and implementation of complex sequences [28]. The PM is generally known to have several basic features of neural organization of motor planning [29]. Our previous study using movement-related cortical potentials during consecutive motor tasks also showed that the peak amplitude in readiness potential was significantly larger under muscle contraction trials than under muscle relaxation trials at the PM, but not at the PPC [18]. Based on these findings, consecutive motor tasks with muscle contraction and relaxation used in the present study might be categorized as complex motor tasks, and neural activity in the PM will be related to performing and controlling forces. In addition, since the peak amplitude of oxy-Hb was significantly more negative at the ipsi- than contralateral hemisphere in the PM under the L-Down condition, muscle relaxation with force control might be sensitively related to neural modulation in the PM, compared with muscle contraction.

As for the PPC, the characteristics of neural activity might differ from the PM. That is, the peak amplitudes in the PPC were significantly more negative in the contra- than ipsilateral hemisphere under the R-Down and L-Down conditions (Fig 6B), showing different oxy-Hb modulation from the PM. It is known that the PPC is related to spatial sense, information navigation and integration, and a sensorimotor interface for visually-guided movements (see reviews, [30, 31]). Some studies also reported that the PPC is critical for sensorimotor tasks, and that it may function in transforming visual information into motor plans [32, 33]. In order to perform our consecutive motor tasks precisely, visually-guided movements with force control are needed, which would be associated with one of the important roles in neural activity of the PPC. We considered that muscle relaxation might be more sensitive to the characteristics of the PPC. Indeed, no significant difference was observed in the changes of oxy-Hb between contra- and ipsilateral hemispheres of the PPC under the R-Up and L-Up conditions (Fig 6A), which was consistent with a previous fMRI study showing no significant differences in BOLD signals between contra- and ipsilateral hemispheres of the PPC during a visually-guided dynamic power-grip paradigm with dominant and non-dominant hands [34].

ANOVAs for peak latencies under the Up and Down conditions showed that peak latencies were significantly later in the ipsi- than contralateral hemisphere at the PPC and PM, but not at the M1 (Table 2). We inferred that the difference was related to transcallosal transfer between left and right motor-related cortical areas via the projection of the corpus callosum. Indeed, a previous study using constrained spherical deconvolution tractography showed that the PM and SMA have extensive interhemispheric connectivity, exhibiting both dense homologous projections and widespread structural relations with every other region in the contralateral motor network [35]. Some previous studies using neurophysiological methods such as magnetoencephalography showed that inter-hemispheric latency between the secondary somatosensory cortices was about 10–20 ms [36, 37]. In the present study, we evaluated the peak latencies of oxy-Hb in fNIRS with a low temporal resolution, compared with the neural response in magnetoencephalography with a high temporal resolution. However, significant differences between hemispheres might reflect transcallosal transfer.

A methodological limitation of the present study is that no sophisticated filter methods were used to account for motion-related artifacts and/or systemic physiological artifacts (e.g., superficial scalp blood flow, heart rate, blood pressure, and breathing rate) which could have confounded our results [38–40]. Hence, in order to improve the quality of fNIRS data, in future studies, sophisticated motion correction techniques and techniques to account for systemic physiological artifacts should be applied.

## Conclusion

The present study showed differences in neural activation, especially in the PM, between right dominant and left non-dominant hands under consecutive motor tasks with muscle contraction and relaxation. These results suggested that the PM of the left hemisphere among right-handed subjects played an important role in muscle contraction and relaxation with force control. The vast majority of studies have focused on muscle contractions, but the present study provides evidence on the underlying neural mechanisms of muscle relaxation as well as contraction in the study field of motor control.
